# Design of Active Fault-Tolerant Control System for Air-Fuel Ratio control of Internal Combustion engine using nonlinear regression-based observer model

**DOI:** 10.1371/journal.pone.0279101

**Published:** 2022-12-15

**Authors:** Turki Alsuwian, Arslan Ahmed Amin, Muhammad Sajid Iqbal, Muhammad Bilal Qadir, Saleh Almasabi, Mohammed Jalalah

**Affiliations:** 1 Department of Electrical Engineering, College of Engineering, Najran University, Najran, Saudi Arabia; 2 Department of Electrical Engineering, FAST National University of Computer and Emerging Sciences, Chiniot Faisalabad Campus, Chiniot, Punjab, Pakistan; 3 School of Engineering & Technology, National Textile University, Faisalabad, Pakistan; 4 Promising Centre for Sensors and Electronic Devices (PCSED), Advanced Materials and Nano-Research Centre, Najran University, Najran, Saudi Arabia; CNR, National Research Council of Italy, ITALY

## Abstract

Internal Combustion (IC) engines are prevalent in the process sector, and maintaining sufficient Air-Fuel Ratio (AFR) regulation in their fuel system is crucial for enhanced engine performance, fuel economy, and environmental safety. Faults in the AFR system’s sensors cause the engine to shut down, hence, fault tolerance is essential. In order to avoid engine shutdown, this paper offers a novel Active Fault-Tolerant Control System (AFTCS) for air-fuel ratio control of an Internal Combustion (IC) engine in a process plant. In the Fault Detection and Isolation (FDI) unit, the proposed AFTCS uses a nonlinear regression-based observer model for analytical redundancy. The suggested system was simulated in the MATLAB / Simulink environment. The proposed system was tested at two different speeds (300 r/min and 600 r/min) and the results show that the system’s response is within the acceptable bound without compromising the stability. The findings also demonstrate the higher fault tolerance capability for sensor defects of the AFR control system, particularly for the MAP sensor (at 300 r/min) in terms of reduced oscillatory response in comparison to the current literature. Compared to the linear regression-based and Genetic Algorithm (GA) based model, the nonlinear regression-based model results in a more accurate estimation of the faulty sensors. The proposed model is also efficient in terms of computation power and response time.

## 1. Introduction

The difference between the actual and expected value of the parameter is called a fault. The ability of a system to continue service under faulty conditions is referred to as fault tolerance [1]. Faults can occur in any real-world system, reducing the system’s reliability and performance [2–4]. Fault-Tolerant Control (FTC) may be applied to increase the reliability of critical systems such as nuclear power plants and airplanes [5]. The system’s performance can be reduced in defective conditions, but failure to some extent may be accepted if stability is assured. Fault-tolerant control systems are generally described as systems that have high reliability [6]. Important industrial processes, such as combustible fuel and gas, petrochemicals, and fertilizers, where production losses cannot be tolerated and consistent system efficiency is vital, are presently using FTC techniques [7, 8]. The active and the passive form are the two main types of FTC [9]. Active Fault-Tolerant Control System (AFTCS) performs the core purpose of identifying and isolating faulty components in the system. The observer theory is used in Fault Detection and Isolation (FDI), where the plant parameter is compared to a predefined normal value to provide a residual [10]. If the residual is below the acceptable limits, it indicates that there are no errors in the machine. An FDI unit declares defective conditions if the residual is found to have exceeded the prescribed threshold. After that, the controller is reprogrammed to fulfill current operating needs.

The process industry frequently uses internal combustion (IC) engines and maintaining sufficient Air-Fuel Ratio (AFR) regulation in their fuel system is essential for improving engine performance, fuel efficiency, and environmental safety. Fault tolerance is necessary because the AFR system’s sensors can malfunction and force the engine to shut down. Analytical redundancy can be used to implement fault tolerance in IC engines. Analytical redundancy’s fundamental tenet is to compare model behavior to actual system behavior [11]. Several estimation techniques are reported in the literature to introduce analytical redundancy in case of sensor failure. The details of those techniques are discussed in section 2. In this paper, we have proposed a nonlinear regression (NLR) based technique to estimate the sensor values in case of a fault. The proposed technique is tested on two different engine speeds (300 r/min and 600 r/min) and encouraging results are obtained. The details of the results are discussed in the results and discussion section. A comparison of the proposed technique with the existing techniques is also included to highlight the effectiveness of the proposed technique.

The rest of the paper is organized as follows: related work available in the literature is discussed in section 2, research methodology is presented in section 3, results and discussion are presented in section 4 followed by the conclusion at the end.

## 2. Literature review

From the literature, we can find that most of the work related to constructing an FDI unit uses Kalman Filters [3], linear Regression [7], Neural Networks [12], Fuzzy Logic [13], and other related techniques. In a nonlinear model of an aero-engine, Kalman filters are employed [14] for fault detection and location (FDL) in the situation of sensor and actuator problems coexisting. The suggested system solely employs hybrid Kalman filters to identify problems, not to estimate sensor values when a defect occurs. Using fuzzy Logic, the bilateral fuzzy adaptive unscented Kalman filter (UKF) is given in [15] to increase the precision of state estimate in the UKF method. In [16], Fuzzy logic is used by FDI to forecast nonlinear functions, and adaptive control is utilized to correct bias and identify actuator failures. As the fuzzy logic-based systems may accept the wrong data and inputs so both the systems proposed in [15] and [16] may produce inaccurate results under some test conditions. If the entire measurements of the data are not available, Ding and Fang [17] proposed an FDI approach that works with occasional observations. In [18], the FDI system was built for the mass air flow (MAF) sensor of the IC engine based on adaptive observers and two ordinary differential equations that describe the intake temperature and pressure dynamics. The experimental implementation of the FDI system allows the continuous operation of the IC engine even in a fault presence.

A recent study [12] proposes a novel adaptive neural network-based fault-tolerant control technique for six-degree-of-freedom nonlinear helicopter dynamics and makes the case for a neural network-based strategy. The suggested method is using the extended Kalman filter (EKF) along with ANN to detect and mitigate various sorts of failures on the helicopter actuators, and the helicopter maintains the required trajectory without interruption. However, the use of EKF may make the proposed technique inefficient in terms of response time. Genetic algorithm-based hybrid FTCS and active FTCS for the AFR control of an IC engine are proposed in [19] and [20] respectively. The proposed systems are providing very accurate sensor values in case of a fault, but the genetic algorithm is very sensitive to the initial conditions, and due to inaccurate initial conditions, the algorithm may converge to the local minima and, hence, cannot provide the optimal solution. For uncertain nonlinear pure-feedback systems with dead-zone actuators and stochastic failures, [21] provides an adaptive fault-tolerant compensation controller. The actuator’s failure mode is represented by a scalar Markovian type of function, and an adaptive backstepping design process is used to create the compensating strategy.

A recent comparative study [22] used GA, NLR, and particle swarm optimization (PSO) to estimate the sensor values in the case of the throttle and MAP sensor fault of an IC gasoline engine. This study is only considering the fixed engine speed of 300 r/min. For a class of stochastic nonlinear systems with stochastic failures and input saturation, [23] examines the issue of command filtering-based event-triggered adaptive fuzzy control. To estimate unknown nonlinear functions and system dynamic changes brought on by stochastic errors, fuzzy logic systems (FLSs) are utilized. To reduce the computational cost, the command filtering design technique is adopted. The average dwell time technique is used to examine the active fault-tolerant control problem for a class of switched nonlinear systems [24], in which neural networks are used to design the controller. The study [25] describes a unified FTC approach based on advanced hardware and analytical redundancies for greater reliability. An ANN-based passive FTCS is proposed in [26] in which a highly nonlinear system of two tank canonical systems is considered and the PFTCS approach is applied to the above-mentioned system. The key benefit of the suggested method is that it incorporates ANN to build the controller, therefore, no exact measurement of faults is necessary. However, previous knowledge of the effect of failures on system performance is required, which makes the system difficult to implement.

From the literature, we can see that for fault detection, most of the work involves Kalman filters, lookup tables, linear regression, machine learning, or artificial neural networks (ANN). Linear regression produces less accurate sensor values [4] for faulty sensors and in [27–29] the authors have shown that the Kalman filters and lookup table methods are inefficient in terms of computational time. The ANN-based approach requires forward propagation and backpropagation because gradient descent has to be repeated many times [30]. This fact makes the ANN slow and to speed up the algorithm, one may use the GPU which is an expensive solution. Hence, we have proposed a nonlinear regression-based AFTCS for highly nonlinear sensors of the AFR system of IC Engine for AFR Control. The proposed technique is efficient in terms of computational power and time without compromising on accuracy.

The fundamental contribution of this study is the development of an NLR-based estimate approach for nonlinear sensors, with the restriction that we have a limited dataset for predicting sensor values in the event of a malfunction. The proposed technique is implemented in such a way that it can estimate the sensor values at two different engine speeds. The simulation results prove the superior fault tolerance performance especially for the MAP sensor in terms of less oscillatory response as compared to existing literature works.

## 3. Research methodology

The proposed AFTCS was created in MATLAB/Simulink utilizing the IC gasoline engine’s experimentally validated AFR model [31]. In this model, the AFR system of the gasoline engine is built based on the findings of Crossley and Cook and was fully validated against dynamometer test data [32]. The mathematical equations used for the model construction are in accordance with the Mean Value Engine Model (MVEM) [33]. Moreover, it gives accurate AFR as found in practical gasoline engines [34]. In this model, an integral feedback ratio control is implemented to maintain the AFR to 14.6 with a reduced steady-state error in normal operation. Fault detection and isolation are performed by the engine control unit built with the state flow diagram in which lookup tables are used to provide estimated values of the faulty sensors for analytical redundancy The primary focus of the paper is the design of the FDI unit with a non-linear regression-based observer model to provide estimated values of the faulty sensors to the engine control unit from the other healthy sensors. The findings of modifying this model to match the appropriate AFTCS architecture for a nonlinear regression-based FDI unit have been presented. The Fault Injection Unit (FIU) sends a fault command to each sensor individually, while the other sensors operate properly. For this project, the engine speed is set first at 300 r/min based on the design speed of the MATLAB model, and if the speed sensor fails, the FDI unit provides the same value to the controller. Since process plant engines normally operate at a constant speed and the design FDI contains a controller with 300 r/min of speed in the event that the speed sensor fails. Moreover, the proposed technique is also tested at 600 r/min, and the stability of the system was determined. It is found that at 600 r/min the AFR is within an acceptable bound, and stability is ensured. Load variations and their effects on speed are not explored in this work because its focus is on creating an NLR-based AFTCS system. In order to extract the MAP and throttle sensors data, MATLAB model lookup tables [32] are employed. Using the provided data, nonlinear correlations between MAP and throttle are developed using the nonlinear regression approach. The FDI unit uses these nonlinear correlations to predict the values of the defective sensors.

### 3.1 Air-Fuel Ratio (AFR) control

**Fig 1:**
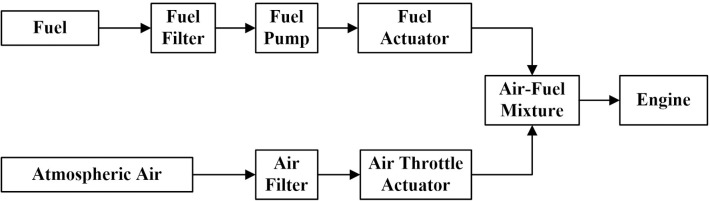
Architecture of Air-fuel system of SI IC engine [35].

An IC engine generates heat and uses air to burn fuel in a combustion chamber. In industrial operations, IC engines are normally employed as prime movers. Chemical energy is turned into mechanical rotation, which powers compressors and alternators. The two types of IC engines are spark ignition (SI) and compression ignition (CI). The compression causes combustion in CI engines, whereas spark plugs cause combustion in SI engines. The AFR determines how frequently air and fuel are mixed in a precise ratio throughout the combustion phase. It’s required for better engine performance, lower fuel consumption, and decreased pollution. Fig 1 depicts the overall design of a SI IC engine’s AFR [34, 35].

The mathematical equation of AFR is:

$$AFR=\frac{m_{air}}{m_{fuel}}$$ (1)

The chemical equation is given as:

$$25O_{2}+2C_{8}H_{18}\to 16CO_{2}+18H_{2}O+Energy$$ (2)

In this equation, the AFR is known as the stoichiometric ratio, and its value for gasoline is 14.6:1. The AFR varies during fuel combustion and can be anywhere from 6:1 and 20:1. In contrast to a lean mixture, a rich mixture has a value that is lower than the stoichiometric ratio. For instance, the 13.7:1 AFR is rich in gasoline whereas the 16.5:1 AFR is lean. Rich and lean mixes are also thought to be harmful to the engine since they degrade the catalyst and reduce power and fuel efficiency. Depending on the kind of gasoline, the value of AFR fluctuates. For instance, the ratio for ethanol is 9:1, for methanol, it is 6.47:1, and for hydrogen, it is 34.3:1 [35].

In order to maintain AFR control of the SI IC engines, four sensors are crucial, which are [31]:

***Manifold Absolute Pressure (MAP) Sensor*:** It gives the controller a precise suction air pressure rating.

***Throttle Sensor*:** It provides the controller with an air throttle position.

***Exhaust Gas Oxygen (EGO) Sensor*:** It is used to assess the oxygen substance in the drained gas and manages the fuel supply to ensure efficient ignition.

***Speed Sensor*:** To measure the rotational speed of the crankshaft of an engine and to provide this speed to the controller, a speed sensor is used.

Because the engine will shut down if these sensors malfunction, fault tolerance is necessary. It would be advantageous to create virtual redundant sensors with a nonlinear response in the FDI unit. Because of this, we approximated the system using the nonlinear regression approach in the AFTCS architecture.

### 3.2 Nonlinear regression

One of the most well-known statistical methods for predictive data mining is regression. Regression is the process of creating a model using training data that enables us to forecast the value of a continuous output variable based on recent input variable values [35]. In statistics, a type of regression analysis called nonlinear regression uses a nonlinear combination of model parameters that rely on one or more independent variables to describe observational data. A method of consecutive approximations is used to match the findings. A nonlinear regression statistical model can be described with the help of the following equation.


Y∼f(X,β) (3)

The aforementioned model is utilized to establish a connection between the vectors of independent variables *X* and *Y*. Unlike linear regression, which has a function *f* that is linear only inside the components of the *β* vector.

The goal of the model is to minimize the sum of square errors. A metric for contrasting *Y* data and the nonlinear (curved) equation used to estimate *Y* is the sum of squares. To obtain it, we compute the difference between each *Y* point in the data set and the fitted nonlinear equation. The next step is to fill up each gap. In the end, combine all the squared figures. If the total of these squared values is minimum, more closely the equation matches the sample’s data points. Several nonlinear functions like Logarithmic functions, exponential functions, power functions, or other similar nonlinear functions are used in nonlinear regression [35]. NLR modeling seeks to graphically track a certain response from a collection of parameters, just like linear regression modeling does. Since the equation is created by a sequence of approximations (iterations) that may result from trial and error, nonlinear models are more difficult to construct than linear models [35].

### 3.3 System modelling

Different dynamic ranges, including air dynamics, fuel dynamics, sensor model, and controller architecture, may be used to categorize the AFR control [36]. Each dynamic’s formulation is provided here. The symbols used during this modeling are given in Table 1.

**Table 1:** List of symbols and their description.

Symbol	Description	Symbol	Description
*m*_*air*_	Mass of Air	${\dot{m}}_{fi}(t)$	Fuel Flow Injection
*m*_*fuel*_	Mass of fuel	${\dot{m}}_{f}(t)$	Fuel Flow into Cylinders
*T*_*in*_	Manifold Input Air Temperature	${\dot{m}}_{fv}$	Vapor Fuel Flow
*P*_*in*_	Manifold Input Air Pressure	${\ddot{m}}_{ff}(t)$	Liquid Mass Fuel Flow
*v*_*in*_	Manifold Input Air Volume	$\dot{\lambda }(t)$	Lambda Sensor
${\dot{m}}_{th}$	Mass Flow Through the Valve	*τ*_*λ*_	Time Delay
${\dot{m}}_{Cyt}$	Mass Flow into Cylinders	*y*_*d*_	Desired Output
R	Gas Constant	*y*	Actual Output
*e*_*x*_	Residual	*u*	Actual Input
*ϕ*_*th*_	Throttle Opening Position	*x*_1_/*x*_2_	State Variables
*C*_*d*_	Discharge Coefficient	*α*/*β*	Parameters of Engine
*S*_*es*_(*ϕ*_*th*_)	True Throttle Opening Position	${\bar{x}}_{1}/{\bar{x}}_{2}$	Estimated Values of Observer Design
*γ*	Heat-ratio of Air	*E*	Root Mean Square Error
*τ*_*f*_	Fuel Vapor Process	*η*	Learning Rate
${\dot{m}}_{fi}(t)$	Fuel Flow Injection	$\bar{y}$	Estimated Output

#### 3.3.1. Air dynamics

The air intake dynamics are described as follows using mass conservation theory and the ideal air-gas hypothesis:

$${\dot{P}}_{in}=\frac{RT_{in}}{v_{in}}({\dot{m}}_{th}-{\dot{m}}_{Cyt})+P_{in}\frac{{\dot{T}}_{in}}{T_{in}}$$ (4)

$${\dot{P}}_{in}=\Psi (\phi_{th},P_{in},T_{in},N_{e})$$ (5)

In Eq 5, *Ne* represents the speed of the engine and the derivative of intake temperature with respect to time is supposed to be zero.

Now, Eq (4) becomes:

$${\dot{P}}_{in}={\dot{k}}_{in}({\dot{m}}_{th}-{\dot{m}}_{Cyt})$$ (6)

$$with\;{\dot{k}}_{in}=\frac{RT_{in}}{v_{in}}$$ (7)

[35] describes how much air passes through the valve and is given as:

$${\dot{m}}_{th}=C_{d}\frac{P_{id}}{\sqrt{{RT}_{id}}}S_{es}(\phi_{th})g\;(P_{r})$$ (8)

*C*_*d*_ the coefficient of discharge. *P*_*id*_ is indicating the overhead load pressure. The load ratio *P*_*r*_ then can be defined as $P_{r}=\frac{P_{in}}{P_{id}}$.

*S*_*es*_(*ϕ*_*th*_) is the throttle opening area and the effective opening throttle area and can be given as:

$$S_{es}(\phi_{th})=\sigma_{1}\{1-\cos\left(\sigma_{2}\phi_{th}+\sigma_{3}\right)\}+\sigma_{4}$$ (9)

Here *g* (*P*_*r*_) is a nonlinear quantity and is given as:

$$g\;(P_{r})=\left\{ \begin{array}{c} \sqrt{\frac{2\gamma }{\gamma -1}}{\left(P_{r}\right)}^{\frac{1}{\gamma }}\sqrt{\left(1-{P_{r}}^{\frac{\gamma -1}{\gamma }}\right)}\;if\;P_{r}>{\left(\frac{2}{\gamma +1}\right)}^{\frac{\gamma }{\gamma -1}}\\ \sqrt{\gamma }{\left(\frac{2}{\gamma +1}\right)}^{\frac{\gamma +1}{2(\gamma -1)}}\;if\;P_{r}\leq {\left(\frac{2}{\gamma +1}\right)}^{\frac{\gamma }{\gamma -1}} \end{array}\right.$$ (10)

The air heat ratio, gamma (γ), is assumed to be 1.4 in this case.

#### 3.3.2 Fuel dynamics

The fuel dynamics can be found in [37] and reported as:

$$\left\{ \begin{array}{c} {\ddot{m}}_{ff}(t)=\frac{1}{\tau_{f}}(-{\dot{m}}_{ff}(t)+x{\dot{m}}_{fi}(t))\\ {\dot{m}}_{fv}=(1-x){\dot{m}}_{fi}(t)\\ {\dot{m}}_{f}(t)={\dot{m}}_{fv}(t)+{\dot{m}}_{ff}(t) \end{array}\right.$$ (11)

Where *τ*_*f*_ is representing the fuel vapor process at some fixed amount of time indicated by (*s*), ${\dot{m}}_{fi}(t)$ is representing the fuel flow injection [*kg*/*s*], the flow of fuel in the cylinders is given by ${\dot{m}}_{f}(t)\;[kg/s],{\dot{m}}_{fv}$ the vapor fuel flow [*kg*/*s*], and ${\ddot{m}}_{ff}(t)$ the liquid mass fuel flow [*kg*/*s*], To produce a more comprehensive model, it is possible to add *ϰ* as a vector dependent on the throttle opening or engine r/min *N*_*e*_ [37]. In our scenario, the second option is more practical.:

$$\tau_{f}(N_{e})=\sigma_{5}{N_{e}}^{{-\sigma }_{6}}$$ (12)

$$\varkappa (N_{e})=\sigma_{7}+\sigma_{8}N_{e}$$ (13)

Where *σ*_5_, *σ*_6_, *σ*_7_, *σ*_8_ are constant parameters. The AFR will now become:

$$\lambda_{cyl}=\frac{{\dot{m}}_{cyl}(t)}{\lambda_{s}{\dot{m}}_{f}(t)}$$ (14)

#### 3.3.3 Sensor model

The expression of the lambda (λ) sensor model can be formed as:

$$\dot{\lambda }(t)=-\frac{1}{\tau_{\lambda }}\lambda (t)+\frac{1}{\tau_{\lambda }}\lambda_{cyl}(t-\tau (N_{e}(t)))$$ (15)

Here *τ*_*λ*_ is the fixed time delay and its value is 0.1 s.

Speed of the engine *N*_*e*_(*t*) and time delay *τ* are related as follows:

$$\tau (N_{e}(t))=\frac{60}{N_{e}(t)}\left(1+\frac{1}{n_{cyl}}\right)$$ (16)

#### 3.3.4 State space representation

The subsequent equation may be utilized to obtain the state-space model.


$$\left[\begin{array}{c} {\dot{x}}_{1}\\ {\dot{x}}_{2} \end{array}\right]=A\left[\begin{array}{c} x_{1}\\ x_{2} \end{array}\right]+B\left[\begin{array}{c} u_{1}\\ u_{2} \end{array}\right]$$ (17)

$$y=C\left[\begin{array}{c} x_{1}\\ x_{2} \end{array}\right]+D\left[\begin{array}{c} u_{1}\\ u_{2} \end{array}\right]$$ (18)

$$\left\{ \begin{array}{c} {\dot{x}}_{1}=f_{1}(.)x_{1}(t)-f_{2}(.)u(t)\\ {\dot{x}}_{2}=-\frac{1}{\tau_{\lambda }}\lambda (t)+\frac{1}{\tau_{\lambda }}\lambda_{cyl}(t-\tau (N_{e}(t))) \end{array}\right.$$ (19)

With *x*_1_(*t*) = *λ*_*cyl*_, *x*_2_(*t*) = *λ*(*t*), and $u(t)={\dot{m}}_{fi}(t)$:

$$f_{1}(.)=-\frac{1}{\tau_{\lambda }(N_{e})}-\frac{{\ddot{m}}_{cyl}}{m_{cyl}(N_{e},P_{in})}$$ (20)

$$f_{2}(.)=\lambda_{s}\frac{\chi (N_{e})}{\tau_{f}(N_{e})}m_{cyl}(N_{e},P_{in})$$ (21)

Bounded as follows: $\underline{f_{i}}\leq f_{i}(∙)\leq \bar{f_{i}}$, for *i*∈{1,2}.

#### 3.3.5 Design of FDI unit

Wang et al. proposed the mathematical model in [38] to formulate the observer’s architecture. The same logic may be used to explain how AFTCS works in state space.


$$\dot{x}=Ax+Bu$$ (22)

y=Cx+Du (23)

$$\dot{\bar{x}}=A\bar{x}+Bu$$ (24)

$$\bar{y}=C\bar{x}$$ (25)

$$\left(\dot{\bar{x}}-\dot{x})=A(\bar{x}-x\right)$$ (26)

$$\left(\bar{y}-y)=C(\bar{x}-x\right)$$ (27)

$$\dot{\bar{x}}=A\bar{x}+Bu+L(\bar{y}-y)$$ (28)

In (28), *L* is indicating the feedback gain.


$$\dot{\bar{x}}-\dot{x}=A(\bar{x}-x)+L(\bar{y}-y)$$ (29)

$$\left(\bar{y}-y)=C(\bar{x}-x\right)$$ (30)

$$\dot{\bar{x}}-\dot{x}=(A+LC)(\bar{x}-x)$$ (31)

$${\dot{e}}_{x}=(A+LC)e_{x}$$ (32)

$$(\bar{y}-y)=Ce_{x}$$ (33)

The FDI unit will not detect a fault until the residual "*e*_*x*_" tends to zero. If the residual (difference) exceeds a certain set point, then the fault is recognized and the FDI unit is utilized to replace the incorrect value with the newly generated estimated value which is obtained from an observer. AFTCS’s complicated structure and sluggish reaction time owing to excessive calculations are two of its key shortcomings [39].

**Lemma 1:** Let us consider the equation for a nonlinear system’s observer design:

$$\dot{\bar{x}}(t)=A\bar{x}+Bu+g(\bar{x,}u,k)+\bar{L}(C\bar{x}-y)$$ (34)

Here the symbols A, B, and C are indicating matrices and “g” is a function of *x*, *u*, and *y*, and $\bar{L}$ is denoting the feedback gain.

Let *e*_*x*_(*t*) be the error

$$e_{x}(t)\hat{=}\bar{x}(t)-x(t)$$ (35)

For a nonlinear system observer, the error equation becomes:

$${\dot{e}}_{x}=(A+\bar{L}C)e_{x}(t)+(g(\bar{x},u,t)-g(x,u,t))$$ (36)

The error *e*_*x*_(*t*) asymptotically approaches 0 if we can obtain a matrix R, X, and scalar μ such that *R* = *R*^*T*^>0 and *μ*>0 in order to meet the linear matrix inequality (LMI):

$$\left[\begin{array}{cc} RA+A^{T}R+XC+C^{T}X^{T}+\mu \lambda^{2}I & R\\ R & -\mu I \end{array}\right]<0$$ (37)

The reliability of each sensor is denoted by R.

The following equation can be used to select the observer gain matrix:

$$\bar{L}=R^{-1}X$$ (38)

We can validate the choice of observer gain matrix by evaluating the following Lyapunov function and proving its derivative to be zero:

$$V(k)=e_{x}^{T}Re_{x}(k)$$ (39)

Next, we need to check $\dot{V}(x)<0\forall x\epsilon D-\{0\}$ as listed below:

$$\dot{V}(k)=e_{X}^{T}(RA+R\bar{L}C+A^{T}R+C^{T}L^{-T}R)e_{x}+2e_{x}^{T}R(g(\bar{x,}u,k)-g(x,u,k))\leq e_{X}^{T}(RA+R\bar{L}C+A^{T}R+C^{T}L^{-T}R)e_{x}+1/\mu \;e_{x}^{T}R^{2}e_{x}+\mu {\left\|g(\bar{x,}u,k)-g(x,u,k)\right\|}^{2}\leq e_{X}^{T}(RA+R\bar{L}C+A^{T}R+C^{T}L^{-T}R)e_{x}+1/\mu \;e_{x}^{T}R^{2}e_{x}+\mu \lambda^{2}{\left\|e_{x}\right\|}^{2}$$

$$=e_{X}^{T}((RA+R\bar{L}C+A^{T}R+C^{T}L^{-T}R)+\mu \lambda^{2}I+1/\mu \;R^{2})e_{x}$$ (40)

Substituting (39) in (40) we get:

$$\dot{V}(k)\leq e_{X}^{T}((RA+R\bar{L}C+A^{T}R+C^{T}L^{-T}R)+\mu \lambda^{2}I+1/\mu \;R^{2})e_{x}$$ (41)

If (41) is satisfied, *e*_*x*_ converges asymptotically to zero.


$$((RA+R\bar{L}C+A^{T}R+C^{T}L^{-T}R)+\mu \lambda^{2}I+1/\mu \;R^{2})<0$$ (42)

Hence, the proof is completed.

**Theorem 1:** The error *e*_*x*_(*t*) approaches to zero exponentially with rate κ/2 if there exist matrices R, X and scalars μ, κ such that *R* = *R*^*T*^>0 *and* μ, k>0 to satisfy the following:

$$\left[\begin{array}{cc} RA+A^{T}R+XC+C^{T}X^{T}+\mu \lambda^{2}I+ⲕR & R\\ R & -\mu I \end{array}\right]<0$$ (43)

$$\bar{L}=R^{-1}X$$ (44)

To prove this, consider (41) and (43) to get

$$\dot{V}(t)\leq -ⲕe_{x}^{T}Re_{x}=-ⲕV(t)$$ (45)

Hence, we can write

$$V(t)\leq e_{x}^{T}V(0)$$ (46)

From (39) we get

$$\lambda_{min}(R){\left\|e_{x}(t)\right\|}^{2}\leq e^{-kt}\lambda_{max}(R){\left\|e_{x}(0)\right\|}^{2}$$ (47)

Where *λ*_*min*_ is the minimum and *λ*_*max*_ is the maximum Eigenvalue of R. As a result, we get the following norm:

‖ex(t)‖≤λmax(R)λmin(R)‖ex(0)‖e−kt/2 (48)

Coming back to the residual equation:

$$r(t)\hat{=}\|C\bar{x}(t)–y(t)\|$$ (49)

r(t)≤λmax(R)λmin(R)C(0)‖ex(0)‖e−kt/2 (50)

$$C(0)\|e_{x}(0)\|\approx \|r(0)\|$$ (51)

Finally, we can come up with the following criteria for fault detection of a sensor fault

r(t){≤λmax(R)λmin(R)‖r(0)‖e−kt2,thereisnofault>λmax(R)λmin(R)‖r(0)‖e−kt2,thereisafault (52)

**Fig 2:**
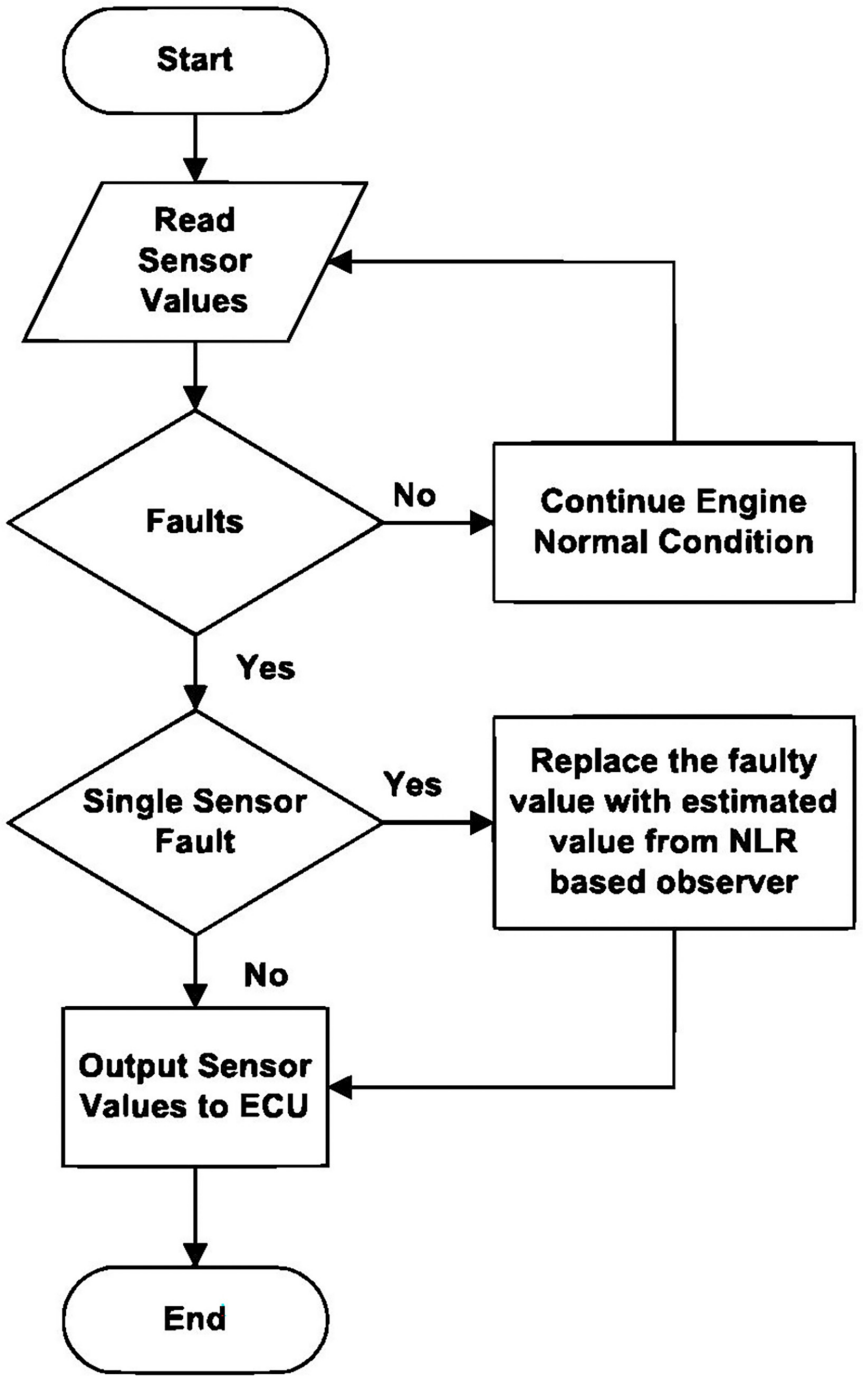
Flowchart of proposed FTCS.

Fig 2 depicts the flowchart of the proposed AFTCS. When the system first begins, it tests the sensor values and calculates the sensor-to-observer value threshold *ξ*. The FDI identifies the defect by computing the residual *e*_*x*_ and compares it to the threshold *ξ* in the following way:

If *e*_*x*_<*ξ*, There is no fault in the sensor.

If *e*_*x*_≥*ξ*, A sensor fault has been found, and the observer output will be used to replace it.

If there is no malfunction, the engine operates as expected. In contrast, the error signal exceeds the threshold (10% absolute) if a single sensor fails. To replace the erroneous sensor value, the FDI unit transmits to the Engine Control Unit (ECU) an approximation value obtained from the observer model based on nonlinear regression. The model’s analytical redundancy is given by the output of the malfunctioning sensor’s approximate simulated value.

This model relies on the assumption that the engine rotates at a constant speed of 300 or 600 r/min, accordingly. Additionally, it was considered that switching and reconfiguring took zero seconds. Practically, the controller calculations will take longer to complete. The study has limitations since it only looks at complete sensor failures, ignoring partial failures, which will be included in subsequent studies. This issue can be caused by a variety of physical issues, including open circuits due to broken wires or faulty connections, and burnout due to any form of short-circuiting that causes the sensor to provide low output.

## 4. Results and discussion

### 4.1 Nonlinear regression analysis

#### 4.1.1 MAP estimation

The fault estimation unit is built utilizing the nonlinear regression approach and data from the model’s lookup tables. No speed measurement is required because the engine’s speed is assumed to remain constant. The throttle and MAP sensor values are selected for this speed. Table 2 shows the lookup table entries for MAP estimates at 300 and 600 r/min.

**Table 2:** Throttle angle and MAP values at 300 and 600 r/min [32].

Throttle Angle Degrees	MAP Value (bar) At 300 r/min	MAP Value (bar) At 600 r/min	Throttle Angle Degrees	MAP Value (bar) At 300 r/min	MAP Value (bar) At 600 r/min
0	0.0913	0.0358	27	0.9643	0.7589
3	0.1136	0.0443	30	0.9752	0.8253
6	0.1907	0.073	35	0.9853	0.8931
9	0.3288	0.1216	46	0.994	0.9553
12	0.5451	0.1904	57	0.9969	0.9766
15	0.7446	0.2806	68	0.9981	0.9855
18	0.8567	0.3944	79	0.9987	0.9898
21	0.9149	0.5343	90	0.9989	0.992
24	0.9463	0.6634			

Nonlinear regression analysis is carried out by using Microsoft Excel Solver Add-in and the nonlinear equation obtained for MAP estimation is given below.


$$y=\left[1-(e^{-ax^{b}})\right]+ce^{-\frac{x}{9}}-de^{\frac{2x-46}{1000}}$$ (53)

Here *y* is the estimated MAP value and *x* is the throttle angle, and *a*, *b*, *c*, and *d* are constants. After running the solver Add-in, the values of unknown constants *a*, *b*, *c*, and *d* are found for 300 and 600 r/min and are given in Table 3 below.

**Table 3:** Values of unknown constants for MAP estimation.

Constant	Speed	
	300 r/min	600 r/min
*a*	0.0027	0.0006
*b*	2.2560	2.3586
*c*	0.1071	0.0647
*d*	0.0114	0.0164

**Fig 3:**
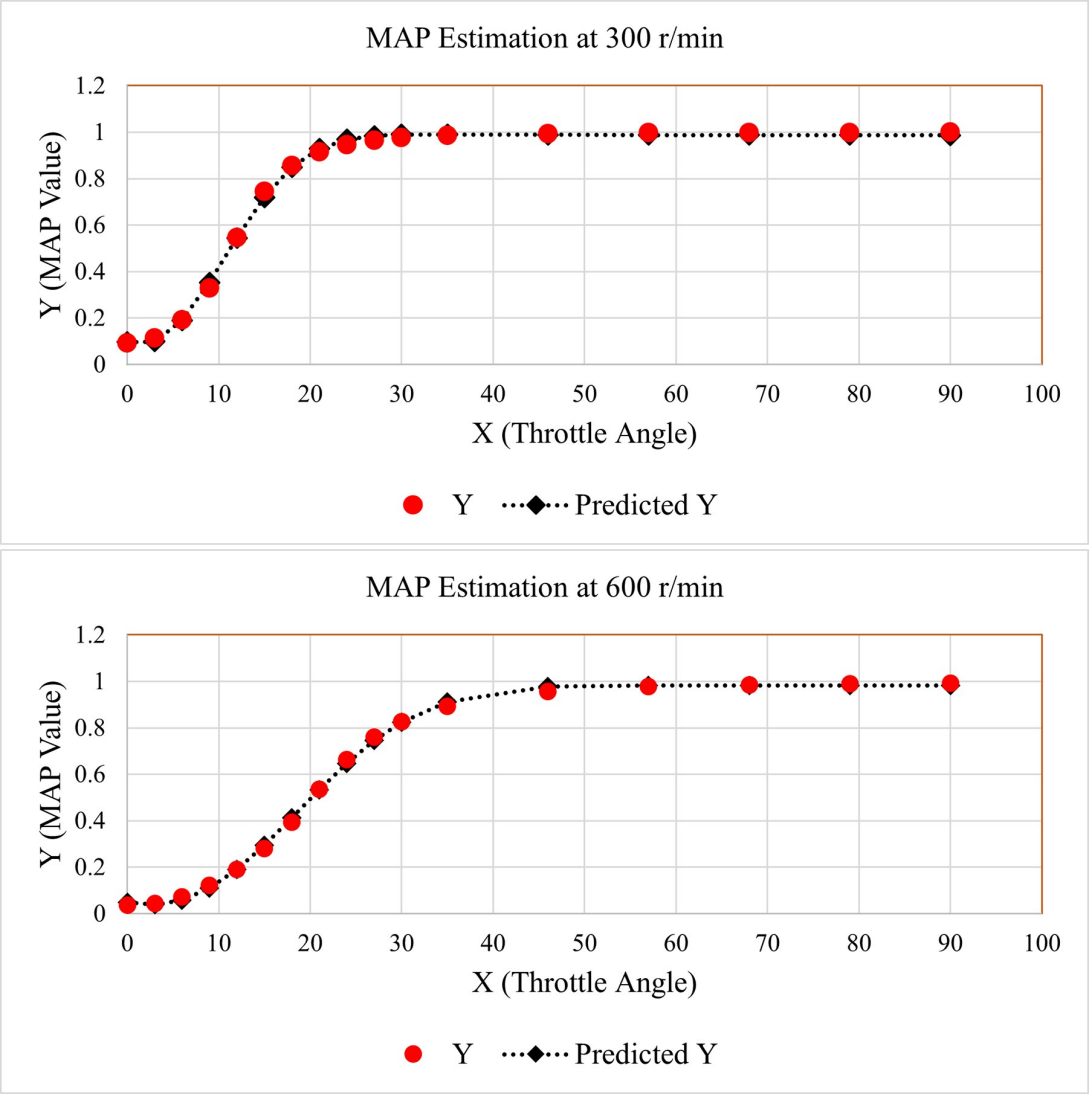
Line fit plots for MAP estimation (a) 300 r/min (b) 600 r/min.

By using the above-mentioned values of unknown constants, the estimated MAP values were generated for both 300 and 600 r/min. For better visualization, the relation between original and estimated MAP values is elaborated by the line fit plot for nonlinear regression, as given below in Fig 3.

It is evident from Fig 3 that the predicted MAP values are very close to the actual values for all the data points. The line fit plots given in Fig 3 prove that the proposed NLR technique is highly accurate.

#### 4.1.2 Throttle estimation

Just like MAP estimation, throttle estimation is also carried out on the data collected from the model’s lookup tables. Speed is again first fixed to 300 r/min and then 600 r/min and nonlinear regression analysis is carried out using Microsoft Excel. Lookup table entries for throttle estimation at 300 and 600 r/min are given in Table 4 below.

**Table 4:** MAP value and throttle angles at 300 and 600 r/min [32].

MAP Value (bar)	Throttle Angle (Degrees) At 300 r/min	Throttle Angle (Degrees) At 600 r/min	MAP Value (bar)	Throttle Angle (Degrees) At 300 r/min	Throttle Angle (Degrees) At 600 r/min
0.05	0	3.7985	0.55	12.0612	21.3303
0.1	1.979	7.8143	0.6	12.704	22.4339
0.15	4.6869	10.3498	0.65	13.4026	23.6495
0.2	6.2586	12.3572	0.7	14.1879	25.0328
0.25	7.4714	14.0618	0.75	15.1073	26.6716
0.3	8.4824	15.5608	0.8	16.2426	28.719
0.35	9.3572	16.9068	0.85	17.7544	31.4829
0.4	10.1308	18.132	0.9	20.0332	35.7286
0.45	10.8244	19.258	0.95	24.4971	44.3714
0.5	11.4521	20.3			

Nonlinear regression analysis is carried out by using Microsoft Excel Solver Add-in and the nonlinear equation obtained for throttle estimation is given below.


y=aln(x)+b (54)

Here *y* is the estimated throttle angle and *x* is the MAP value. *a* and *b* are constants. After running the solver Add-in, the values of *a* and *b* are found, and a line fit plot was obtained. But the above equation did not provide the best estimation. For this equation, the error term at the first and last two entries was very high. So, to fine-tune the fitting curve, a polynomial term was added to the above equation. And the final nonlinear equation for the throttle estimation is given below.


$$y=aln(x)+bx^{3}-cx^{2}+dx$$ (55)

Here *y* is the estimated throttle angle and *x* is the MAP value and *a*, *b*, *c*, and *d* are constants. After running the solver Add-in, the values of unknown constants *a*, *b*, *c*, and *d* are found for 300 and 600 r/min and are given in Table 5 below.

**Table 5:** Values of unknown constants for throttle estimation.

Constant	Speed	
	300 r/min	600 r/min
*a*	1.0183	0.2505
*b*	73.292	128.818
*c*	104.449	176.901
*d*	57.774	96.1621

**Fig 4:**
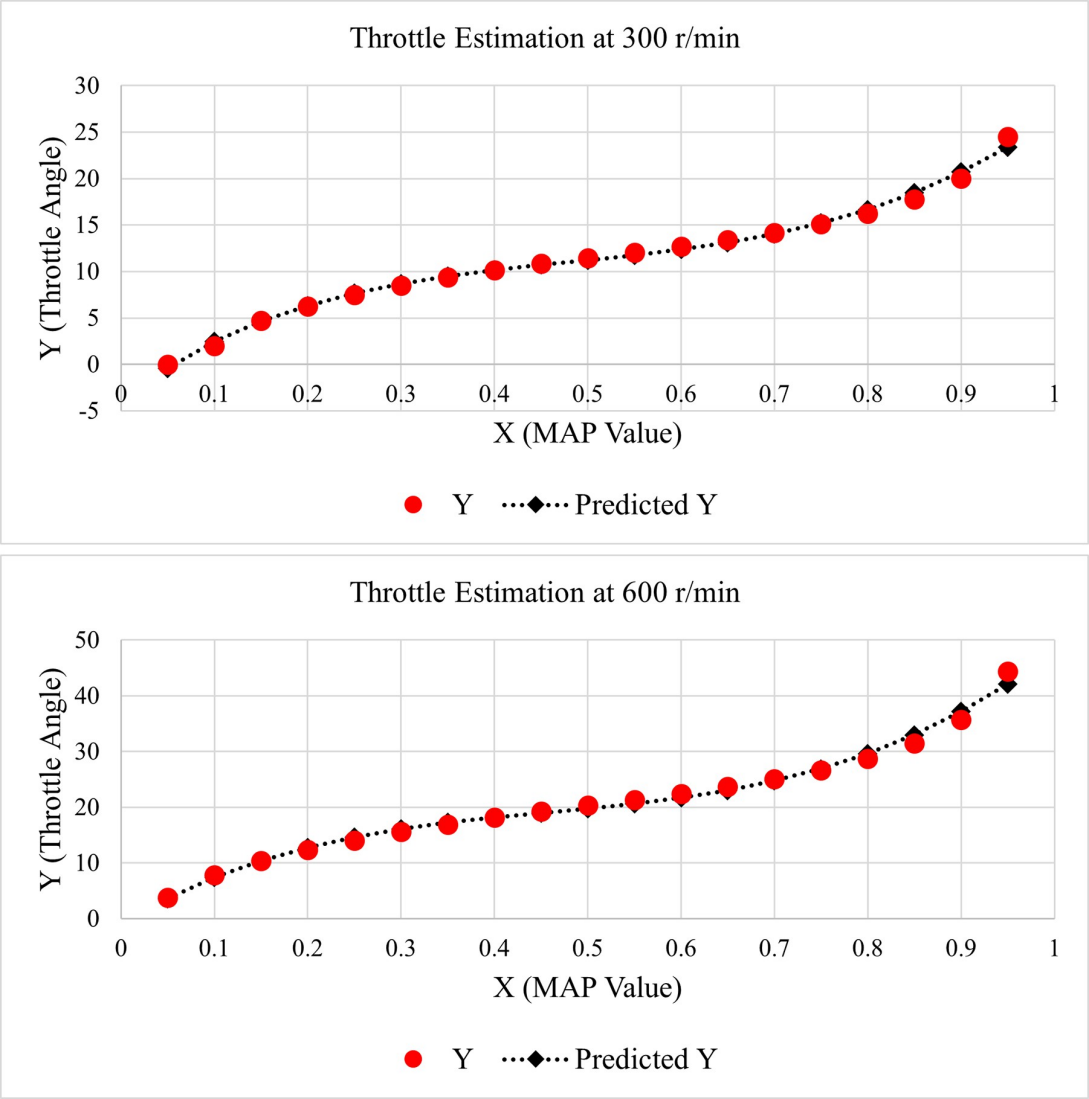
Line fit plot for throttle estimation (a) 300 r/min (b) 600 r/min.

By using the above-mentioned values of unknown constants, the estimated throttle values were generated for both 300 and 600 r/min. For better visualization, the relation between original and estimated throttle values is elaborated by the line fit plot for nonlinear regression, as given below in Fig 4.

It is evident from Fig 4 that the predicted throttle values are very close to the actual values for all the data points. The line fit plots given in Fig 4 prove that the proposed NLR technique is highly accurate for throttle estimation as well.

### 4.2 MATLAB simulation results

**Fig 5:**
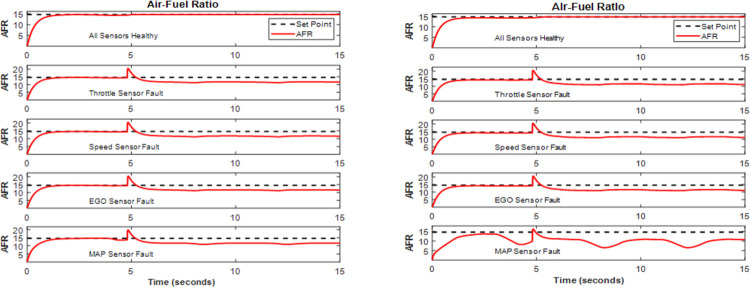
AFTCS performance for AFR control of IC engine (a) at 300 r/min (b) at 600 r/min.

In a MATLAB IC Gasoline Engine model, the suggested AFTCS for the AFR controller was implemented for two different engine speeds (300 r/min and 600 r/min). The FDI block is used to identify, separate, and replace invalid parameters with their appropriate values. The observer model of the FDI unit was developed using NLR connections. The NLR observer develops a new estimated value based on input from the other functional sensors in the FDI unit, which is sent to the controller during the AFTCS phase. The FIU injects the flaw into each sensor individually, maintaining the health of the other sensors. The mixture AFR ratio is held constant at 14.6 under normal circumstances but falls to 11.7 during fault situations (rich mixture). The system stays stable despite the degradation, which is compatible with the AFTCS design philosophy [40]. Fig 5 depicts how the proposed AFTCS operates under normal and abnormal conditions.

We can notice from Fig 5(A) that at 300 r/min the AFR is initially set at 14.6 and when there is a fault in any sensor, the AFR changes for a very short instance of time and then sets to 11.6 while ensuring the system stability. From Fig 5(B) we conclude that at 600 r/min the AFR is maintained at 14.6 for healthy sensors and in case of a fault the AFR changes to 11.6. For the MAP sensor there are more oscillations in AFR, but we can notice that the value of AFR is within the acceptable bound for the entire duration of time which will ensure the system’s stability.

### 4.3 Reliability analysis

In this section, the probabilistic reliability analysis has been carried out for our proposed AFTCS model, as discussed in [22]. Let *R* represents the reliability of each sensor, the probability of failure would be as follows:

$$P_{CF}=1-R$$ (56)

where *P*_*CF*_ represents the probability of component failure.

Without fault tolerance, the engine will get shut down due to a fault in any of the four sensors.

Let *P*_*SF*_ represents the probability of system failure and *R*_*System*_ represents the reliability of the overall system, then we have

$$P_{SF}=4(1-R)$$ (57)

$$R_{System}=1–P_{SF}$$ (58)

$$R_{System}=1–4(1-R)$$ (59)

$$If\;R=0.9\;\mathrm{then}\;R_{System}=1–4(1-0.9)=0.6$$

$$R_{System}=60\%$$

With the proposed AFTCS, the engine does not get shut down in case of a fault in any sensor one at a time. However, the engine would get shut down in case of failure of any two sensors at a time. Therefore,

$$P_{SF}={}_{2}^{4}C(1-R)(1-R)$$ (60)

$$P_{SF}=6{\left(1-R\right)}^{2}$$ (61)

$$R_{System}=1–P_{SF}$$ (62)

$$R_{System}=1–6{\left(1-R\right)}^{2}$$ (63)

$$If\;R=0.9\;then\;R_{System}=1–6{\left(1-0.9\right)}^{2}=0.94$$

$$R_{System}=94\%$$

Thus, the reliability of the system improves by 34% with the proposed AFTCS for sensor faults.

Hence, we conclude that the proposed AFTCS improves the system’s reliability.

### 4.4 Comparison with existing approaches

In this section, the proposed technique is compared with some existing AFTCS approaches for IC engines operating at 300 r/min. Three approaches are using ANN [10], linear regression (LR) [7], and genetic algorithm (GA) [20]. In the ANN-based approach, the authors have excluded some points during the training stage as they were acting as outliers so a better comparison can be made with linear regression and genetic algorithm-based approach reported in [7] and [20] respectively.

#### 4.4.1 MAP sensor comparison

To compare the proposed technique with LR and GA-based approaches, the estimated MAP values and actual MAP values are recorded, and the percentage error is calculated against each data point. As the MAP values are very small, that is why we have used the percentage error instead of the mean square error. Table 6 summarizes the actual and estimated MAP values along with the percentage error in the proposed, LR, and GA-based techniques.

**Table 6:** Actual and estimated MAP values and percentage errors.

Throttle Angle Degrees	Actual MAP Value bar	Proposed Technique		LR [7]		GA [20]	
		Estimated Value bar	% Error	Estimated Value bar	% Error	Estimated Value bar	% Error
0	0.091	0.096	5.25	0.458	401.8	0	100.00
3	0.114	0.098	13.73	0.485	326.2	0.115	1.10
6	0.191	0.189	0.82	0.512	168.3	0.282	47.70
9	0.329	0.352	7.17	0.539	63.84	0.447	36.04
12	0.545	0.545	0.11	0.566	3.78	0.592	8.65
15	0.745	0.720	3.35	0.593	20.39	0.710	4.69
18	0.857	0.849	0.88	0.620	27.66	0.800	6.65
21	0.915	0.928	1.46	0.647	29.31	0.866	5.38
24	0.946	0.968	2.30	0.674	28.80	0.912	3.60
27	0.964	0.984	2.08	0.701	27.32	0.944	2.11
30	0.975	0.989	1.47	0.728	25.37	0.965	1.04
35	0.985	0.990	0.50	0.773	21.56	0.985	0.05
46	0.994	0.989	0.54	0.872	12.28	0.998	0.40
57	0.997	0.988	0.90	0.971	2.60	1.000	0.29
68	0.998	0.988	1.06	1.070	7.21	1.000	0.19
79	0.999	0.987	1.15	1.169	17.07	1.000	0.13
90	0.999	0.987	1.20	1.268	26.96	1.000	0.11

**Fig 6:**
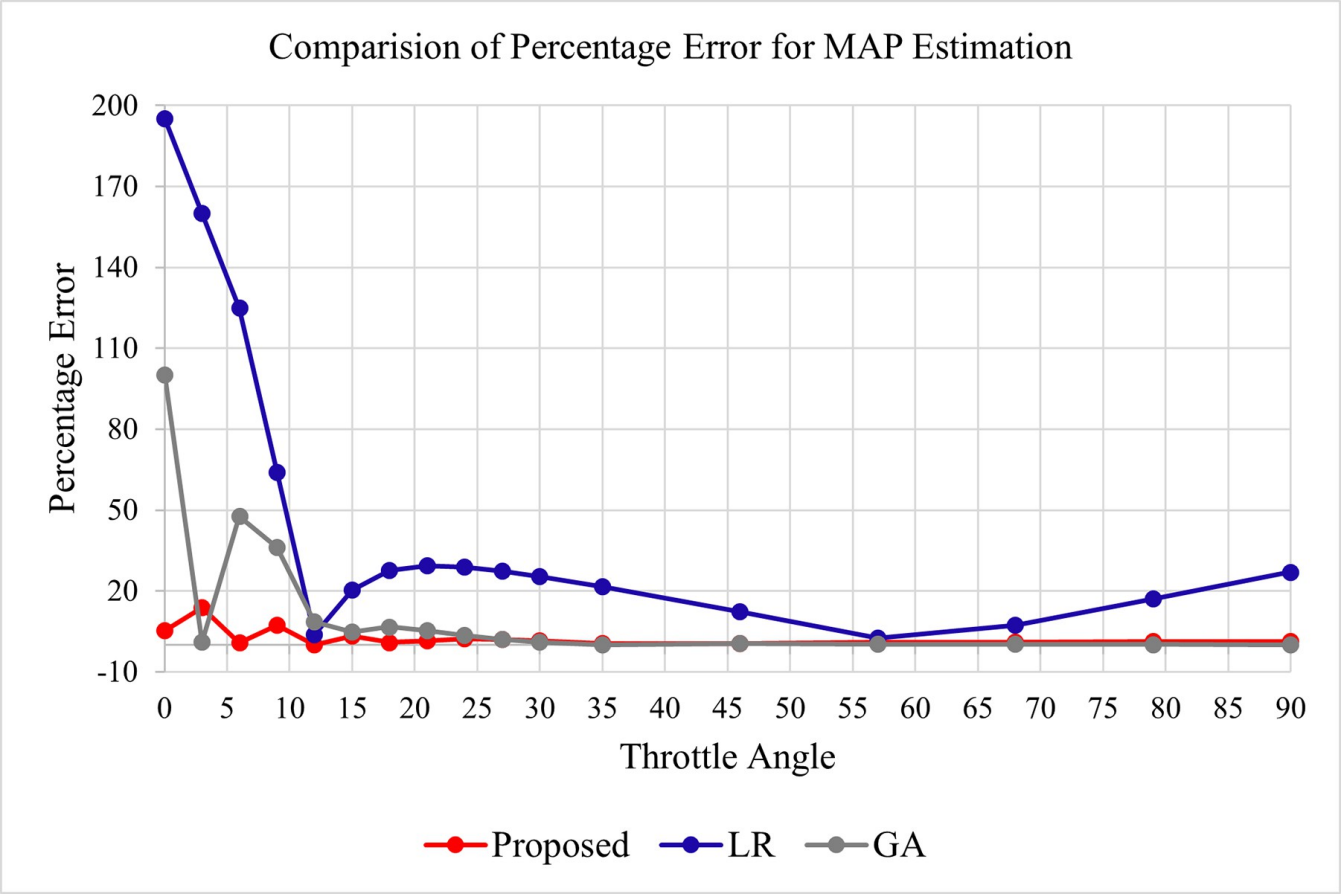
Percentage error comparison for MAP estimation in proposed, LR, and GA.

A graphical representation of percentage error for MAP estimation in the proposed, LR, and GA-based techniques is given in Fig 6 below.

It can be noticed from Fig 6 that the percentage error in the proposed technique is very close to 0 against all the throttle values which indicates the accuracy and consistency of the proposed technique. On the other hand, the error percentage in LR based approach is very high. In the GA-based approach, the error is very negligible for the throttle values greater than 300 but before 300, the percentage error is very high which shows although the GA-based approach is producing a low mean square error, the error percentage at some points is very high.

#### 4.4.2 Throttle sensor comparison

To compare the proposed technique with LR and GA-based approaches, the estimated throttle values and actual throttle values are recorded, and the percentage error is calculated against each data point. Table 7 summarizes the actual and estimated throttle values along with the percentage error in the proposed, LR, and GA-based techniques.

**Table 7:** Actual and estimated throttle values and percentage errors.

MAP Value bar	Actual throttle Value degrees	Proposed Technique		LR [7]		GA [20]	
		Estimated Value degrees	% Error	Estimated Value degrees	% Error	Estimated Value degrees	% Error
0.05	0	-0.414	40.00	1.994	199.00	-0.115	11.00
0.1	1.979	2.461	24.38	3.040	53.59	1.951	1.44
0.15	4.687	4.632	1.18	4.085	12.85	3.780	19.36
0.2	6.259	6.324	1.05	5.130	18.03	5.365	14.28
0.25	7.471	7.649	2.38	6.175	17.35	6.708	10.22
0.3	8.482	8.685	2.38	7.221	14.87	7.819	7.82
0.35	9.357	9.499	1.52	8.266	11.66	8.719	6.82
0.4	10.131	10.155	0.24	9.311	8.09	9.436	6.86
0.45	10.824	10.713	1.03	10.356	4.32	10.008	7.55
0.5	11.452	11.230	1.94	11.402	0.44	10.481	8.48
0.55	12.061	11.765	2.46	12.447	3.20	10.912	9.53
0.6	12.704	12.374	2.60	13.492	6.21	11.366	10.53
0.65	13.403	13.113	2.16	14.538	8.47	11.916	11.09
0.7	14.188	14.038	1.06	15.583	9.83	12.646	10.87
0.75	15.107	15.205	0.65	16.628	10.07	13.647	9.67
0.8	16.243	16.670	2.63	17.673	8.81	15.021	7.52
0.85	17.754	18.488	4.13	18.719	5.43	16.878	4.94
0.9	20.033	20.715	3.41	19.764	1.34	19.337	3.48
0.95	24.497	23.407	4.45	20.809	15.05	22.526	8.05

**Fig 7:**
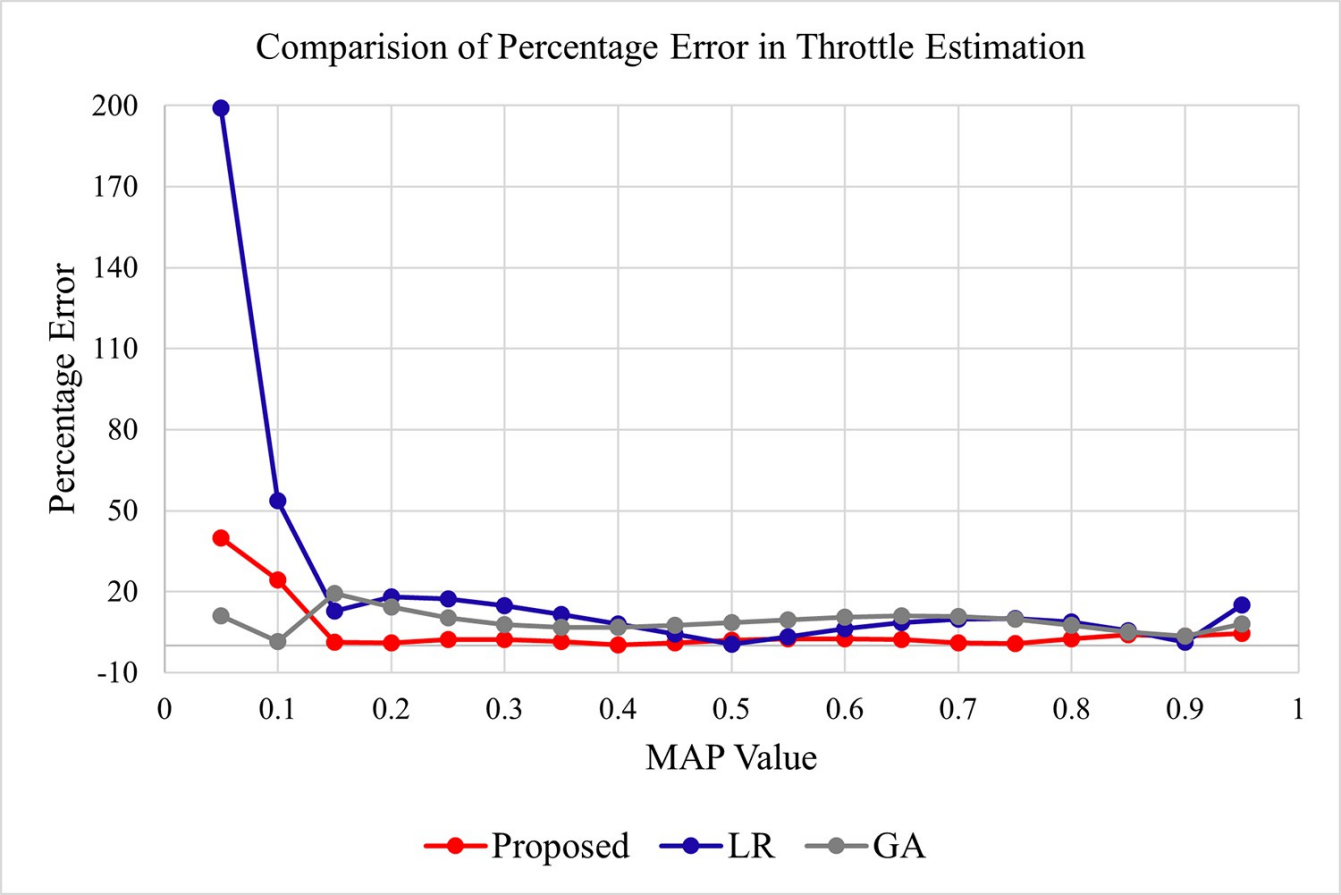
Percentage error comparison for throttle estimation in proposed, LR, and GA.

A graphical representation of percentage error for throttle estimation in the proposed, LR, and GA-based techniques is given in Fig 7 below.

It is evident from Fig 7 that the percentage error against each data point in the proposed technique is very low except first two points. Whereas in LR the error is very high and for GA the error is in the range of 15%. The above graph also indicates that the proposed technique is producing the estimated throttle values with high accuracy and consistency.

### 4.5 Complexity analysis

During this study, we used the nonlinear regression-based model to predict the MAP and throttle estimation and one major objective was to develop a method for the devices having computational power constraints and time-critical applications. The available techniques producing relatively accurate results in the literature mostly use LTs, KF, ANN, or GA. It is already discussed in [27–29] that the KFs and LTs are highly inefficient in terms of computational time and power. If we consider the ANN, the two primary processes in ANN training are forward propagation and backpropagation, and the complexity of both operations is a fifth-degree polynomial [41]. Furthermore, backward propagation has a low convergence rate and poor generalization capability, making it computationally inefficient [41]. In GA, there is no proper way to determine the time complexity and the cost function itself decides the complexity of the problem [42]. In this study, the models used for prediction are exponential and of 4^th^-degree polynomial, hence, the time complexity in using GA will be very high. If we consider a regression-based model with n number of training examples and m number of features then the Train Time Complexity will be equal to O(nm^2^ + m^3^) and Test Time Complexity will be equal to O(m) [30]. In this problem, we are having only two features in our regression problem and, hence, the total time complexity will become O(n), which is very much less than the ANN and GA. Hence, we conclude that the proposed technique is efficient in terms of response time and computational time and can be used with time-critical applications.

## 5. Conclusions

In this article, an advanced AFTCS for AFR control of the IC engine was proposed in order to achieve greater reliability and prevent costly shutdowns due to sensor faults. In the proposed AFTCS, the FDI unit was constructed using an observer unit based on nonlinear regression. The model was developed in MATLAB/Simulink and evaluated for several sensor problems and two engine speeds. The findings demonstrated higher fault tolerance capability for sensor malfunctions of the AFR control system, particularly the MAP sensor, in terms of reduced oscillatory response as compared to previous literature. A comparison of the proposed nonlinear regression-based model is presented with the linear regression and GA-based techniques. The results showed that the proposed technique has a very low percentage error on all data points as compared to techniques available in the literature. It is also proved that the proposed model is efficient in terms of computation power and response time.

Since we have studied full-type failures of the sensors as a limited scenario of research, future work may incorporate the AFTCS design for partial-type faults of the sensors in conjunction with experimental investigation, such as hardware-in-the-loop testing. Another direction is to consider the time-varying delays for the fault-tolerant AFR controller design.

## Supporting information

S1 Data (ZIP)
